# Does Aphid Infestation Interfere with Indirect Plant Defense against Lepidopteran Caterpillars in Wild Cabbage?

**DOI:** 10.1007/s10886-017-0842-z

**Published:** 2017-04-12

**Authors:** Yehua Li, Berhane T. Weldegergis, Surachet Chamontri, Marcel Dicke, Rieta Gols

**Affiliations:** Laboratory of Entomology, Wageningen University, P.O. Box 16, 6700 AA Wageningen, The Netherlands

**Keywords:** *Brassica oleracea*, *Diadegma semiclausum*, Indirect defense, *Mamestra brassicae*, *Microplitis mediator*, Multiple herbivory, Natural enemies, Parasitoid behavior, *Plutella xylostella*, Plant volatiles

## Abstract

Attraction of parasitoids to plant volatiles induced by multiple herbivory depends on the specific combinations of attacking herbivore species, especially when their feeding modes activate different defense signalling pathways as has been reported for phloem feeding aphids and tissue feeding caterpillars. We studied the effects of pre-infestation with non-host aphids (*Brevicoryne brassicae*) for two different time periods on the ability of two parasitoid species to discriminate between volatiles emitted by plants infested by host caterpillars alone and those emitted by plants infested with host caterpillars plus aphids. Using plants originating from three chemically distinct wild cabbage (*Brassica oleracea*) populations, *Diadegma semiclausum* switched preference for dually infested plants to preference for plants infested with *Plutella xylostella* hosts alone when the duration of pre-aphid infestation doubled from 7 to 14 days. *Microplitis mediator*, a parasitoid of *Mamestra brassicae* caterpillars, preferred dually-infested plants irrespective of aphid-infestation duration. Separation of the volatile blends emitted by plants infested with hosts plus aphids or with hosts only was poor, based on multivariate statistics. However, emission rates of individual compounds were often reduced in plants infested with aphids plus hosts compared to those emitted by plants infested with hosts alone. This effect depended on host caterpillar species and plant population and was little affected by aphid infestation duration. Thus, the interactive effect of aphids and hosts on plant volatile production and parasitoid attraction can be dynamic and parasitoid specific. The characteristics of the multi-component volatile blends that determine parasitoid attraction are too complex to be deduced from simple correlative statistical analyses.

**Electronic supplementary material:**

The online version of this article (doi:10.1007/s10886-017-0842-z) contains supplementary material, which is available to authorized users.

## Introduction

Natural enemies of herbivorous insects must find their hosts or prey in habitats that are often structurally and chemically heterogeneous (Meiners 2015; Schoonhoven et al. 2005). Plant volatiles are particularly important during foraging behavior of natural enemies of insect herbivores (Dicke and Baldwin 2010; Hare 2011; Heil 2008). Plant volatiles that are produced in response to herbivory, also referred to as herbivore-induced plant volatiles (HIPVs), can be reliable cues in host-location behaviour by parasitoids of insect herbivores (Mumm and Dicke 2010). Moreover, HIPVs may play an important role in structuring plant-associated insect communities as they mediate multitrophic interactions between plants, herbivores and their carnivorous natural enemies (Dicke and Baldwin 2010; Hare 2011; Poelman et al. 2012; Vet and Dicke 1992).

Herbivory by multiple species is the norm in nature. This may affect the induction of HIPVs and as a result the attraction of natural enemies of insect herbivores (De Rijk et al. 2013; Dicke et al. 2009; Ponzio et al. 2013). Reviewing the existing literature, De Rijk et al. (2013) found that when given the choice between volatiles emitted by plants infested with hosts plus non-hosts vs. plants infested with hosts alone, roughly 50% of the insect carnivores investigated did not discriminate between the two sources of volatiles, 25% preferred volatiles emitted by plants infested with hosts alone and an equal percentage preferred dually-infested plants. Plant volatile biosynthesis in response to insect herbivory is initiated by a combination of mechanical damage and elicitors from oral secretions of the herbivores (Bonaventure et al. 2011; Dicke 2009; Mumm and Dicke 2010; Turlings et al. 1990). The composition of the HIPV blend depends on the identity of the attacking herbivore and is also highly plant-species specific (Agbogba and Powell 2007; Dicke et al. 2003; Turlings et al. 1998). However, many of the blend components are produced by a wide range of plant species (Mumm and Dicke 2010). Different herbivore species attacking the same host plant may induce subtle differences in volatile profiles, even when the herbivores belong to the same feeding guild (De Moraes et al. 1998; Delphia et al. 2007; Dicke et al. 2003; Turlings et al. 1998). Thus, the effect of multiple herbivory on the behavioral response of parasitoids to HIPVs depends on the specific combination of host and non-host herbivores (De Rijk et al. 2013; Dicke 2009; Erb et al. 2010; Rodriguez-Saona et al. 2003; Takabayashi et al. 2006; Zhang et al. 2013; Zhang et al. 2009).

The production of HIPVs is controlled by different plant signal-transduction pathways regulated by the key phytohormones jasmonic acid (JA) and salicylic acid (SA) (Arimura et al. 2009; Erb et al. 2012; Heil and Ton 2008). Different types of attack often trigger different signalling pathways in the plant (Dicke et al. 2009). Phloem-feeding insect herbivores, such as aphids, mainly activate SA-dependent responses, whereas leaf-chewing herbivores, such as lepidopteran caterpillars, predominantly trigger JA-dependent responses (Moran and Thompson 2001; Thaler et al. 2012; Zarate et al. 2007). When two herbivore species attack the same plant, these two signalling pathways interact through crosstalk (Koornneef and Pieterse 2008; Thaler et al. 2012). The effect of JA-SA crosstalk on HIPV production and parasitoid attraction to plants infested with two different herbivore species has been shown to be variable (De Rijk et al. 2013; Dicke et al. 2009; Engelberth et al. 2001; Howe and Jander 2008; Ponzio et al. 2013). For example, infestation with phloem-feeding whiteflies (*Bemisia tabaci*) was shown to perturb the JA-mediated plant volatile emission and parasitoid attraction in response to leaf-chewing herbivory (Rodriguez-Saona et al. 2003; Zhang et al. 2013). Contrastingly, the parasitoid *Cotesia marginiventris* showed enhanced attraction to plants simultaneously infested with aphids (*Macrosiphum euphorbiae*) and caterpillars (*Spodoptera exigua*), compared to plants only infested with caterpillars (Rodriguez-Saona et al. 2005).

The temporal dynamics of induction of the signal-transduction pathways regulating HIPV emission differ (Dicke 2009). Metabolic changes in plants in response to chewing herbivores, including the emission of HIPVs, generally occur within hours to one or two days after feeding damage (Dicke et al. 2009; Kunert et al. 2002). The response of plants to the more subtle damage caused by aphid feeding often takes much more time (Dicke 2009; Guerrieri and Digilio 2008; Moran and Thompson 2001; Schmelz et al. 2003). The specific temporal dynamics of defense-pathway induction may have consequences for the response of a plant to two herbivores that attack the plant separated in time (Mouttet et al. 2013). For example, the suppression of JA-responsive gene expression and reduction in JA concentration in aphid-infested *Brassica oleracea* plants was only found to occur six days after aphid attack (Soler et al. 2012).

The objective of this study was to investigate whether volatile-mediated foraging behavior of parasitoids attacking caterpillars of insect herbivores is affected when plants are infested with aphids for two different time periods before the host caterpillars are introduced onto the plant. We compared the behavioral response of two parasitoid species towards volatiles emitted by plants infested with hosts and aphids versus plants infested with hosts alone in a Y-tube olfactometer. HIPV blends produced by different genotypes and cultivars of the same species also display quantitative and/or qualitative variation with concomitant effects on parasitoid attraction (Köllner et al. 2008; Lou et al. 2006; Poelman et al. 2009). To account for this intra-specific variation in HIPV production, we used wild cabbage (*Brassica oleracea* L., Brassicaceae) plants that originated from three wild populations in Dorset, UK, and are known to differ in volatile and non-volatile secondary chemistry (Gols et al. 2011; Harvey et al. 2011). We hypothesized that the duration of aphid infestation and as a result of this, their density, would interfere with the caterpillar-induced volatile production. In addition, we collected and analyzed the volatiles emitted by the plants exposed to the various aphid and caterpillar treatments to assess the underlying chemical basis of the observed behavioral responses.

## Methods and Materials

### Plants and Insects

The major differences in HIPV blend characteristics between the wild cabbage populations used in this study and cultivated cabbage are overall reduced volatile emission and the presence of glucosinolate hydrolysis products (Gols et al. 2011). Glucosinolates are secondary metabolites that are typically produced by brassicaceous plant species and concentrations of these compounds also differ among the three selected populations (Harvey et al. 2011). Seeds of the three wild cabbage (*B. oleracea*) populations originated from sites known as Winspit (50°35′N, 2°02′W), Old Harry (50°38′N, 1°55′W) and Kimmeridge (50°36′N, 2°07′W), abbreviated to WIN, OH and KIM, respectively. Seeds were sown one week before transplanting seedlings into individual pots (1.5 l) containing potting soil (Lentse potgrond no. 4; Lent, The Netherlands). Plants were grown in a greenhouse at 22 ± 3 °C, 50–70% relative humidity [RH], and a light:dark regime [L:D] of 16:8 h. Plants were watered every two days until they were three weeks old, and watered daily hereafter.

The two caterpillar and parasitoid species used in this study differ in their degree of host specialisation. As volatile cues used during foraging behavior may differ between parasitoid species depending on their host range and the food-plant range of the hosts, the effects of pre-infestation with aphids on volatile-mediated foraging may vary as well (Steidle and Van Loon 2003). We selected these two larval endoparasitoid species to increase the potential for variation in parasitoid responses to HIPVs, and not to compare the difference in response of specialist and generalist parasitoids as this would require testing of a range of parasitoid species differing in dietary breadth. *Diadegma semiclausum* Hellén (Hymenoptera: Ichneumonidae) is a specialist endoparasitoid of *Plutella xylostella* (Lepidoptera: Plutellidae) caterpillars which primarily feed on brassicaceous plant species. *Microplitis mediator* Halliday (Hymenoptera: Braconidae) is a generalist endoparasitoid that can attack larval stages of several noctuid species including *Mamestra brassicae* L. (Lepidoptera: Noctuidae). This herbivore is a generalist and a pest on many crop plant species, especially cabbage crops. The aphid *Brevicoryne brassicae* L. (Hemiptera, Aphididae), which is also a specialist on brassicaceous plant species was used to pre-infest the plants before caterpillar hosts were introduced. All insects were originally collected from cabbage fields in the vicinity of Wageningen University and all cultures were maintained on Brussels sprouts plants (*B. oleracea* L. var. gemmifera cv. Cyrus) in a greenhouse or a climate room at 22 ± 2 °C, 60–70% RH and 16:8 h L:D photo regime.

The two parasitoid species were reared on plants infested with host caterpillars until the parasitoids had completed their immature development and pupated. Newly emerged *D. semiclausum* and *M. mediator* adults were collected and transferred to clean insect cages and allowed to mate. Adult wasps were kept in a climate cabinet at 25 ± 1 °C, and L16:D8 photo regime and were provided with honey and 6–10% sugar water as a food source. Both wasp species were considered naïve as plant material had been removed from the cage before the wasps eclosed and wasps had no previous experience with hosts. Female wasps that were used in the experiments were 2–6 (*D. semiclausum*) or 3–7 (*M. mediator*) days old.

### Plant Treatments

When plants were four weeks old, 20 adult aphids (*B. brassicae*) were introduced on the first fully expanded leaf of plants from the three populations and were allowed to feed and reproduce for 7 or 14 days prior to testing in a Y-tube olfactometer (see below). The infested plants were covered with nylon nets (48 × 60 cm, Bugdorm, Taiwan) to prevent cross-contamination. Approximately 24 h before the behavioral experiments, 10 *P. xylostella* caterpillars (L2) or 30 one-day-old *M. brassicae* caterpillars were introduced on the same leaf as where the aphids had been introduced or on a comparable leaf when plants only received a host-caterpillar treatment. The herbivores remained on the plant when used in the bioassay and during volatile trapping to ensure continuation of volatile production.

### Bioassay

The experiment was conducted in a laboratory at 22 ± 2 °C in a Y-tube olfactometer consisting of a Y-shaped glass tube (diameter 3.5 cm, stem length 20 cm, arm length 10 cm). Two glass vessels (35 l) containing an odor source (intact plants) were connected to the arms of the Y-tube olfactometer using Teflon tubes (for details see Takabayashi and Dicke 1992). The instrument was illuminated from above with fluorescent lights at an intensity of 30–35 μmol photons m^−2^ s^−1^. Pressurized air was filtered through activated charcoal and led into each of the two glass vessels at 2 l min^−1^. The experiment started with the release of a wasp at the base of the Y-tube. Each wasp was observed for a maximum of 10 min, and a choice was recorded when the wasp reached the middle of either arm and remained in that arm for at least 15 s. When the wasp did not make a choice within 10 min, a ‘no choice’ behavioral response was recorded. Each wasp was used only once. One experimental replicate consisted of one pair of cabbage plants tested with 8–10 responding female parasitoids.

First, we conducted a pilot test to examine whether both wasp species discriminated between host-infested (24 h) and uninfested plants. This experiment was conducted with both wasp species with plants from the WIN population only and was repeated five times, i.e. 5 sets of plants were used, each tested with 8–10 responding female wasps.

**Fig. 1:**
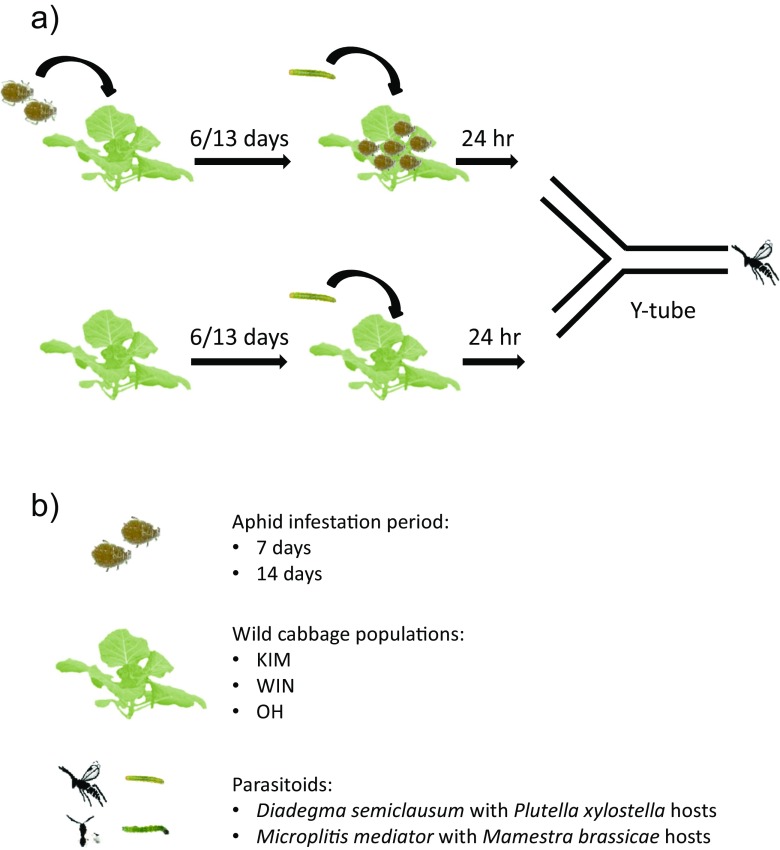
Experimental design **a** and investigated factors **b**. In the Y-tube bioassay each herbivore treatment combination was tested with 8–10 parasitoids and replicated eight times with a new set of plants. The relative preference for dually-infested plants in each replicate was the response variable in the statistical analysis. See also Material and Methods for further details

We then continued with the main experiment in which we compared the response of both parasitoid species to plants infested with hosts alone and plants dually infested with hosts and aphids. The pair-wise comparisons were conducted with plants from the same population. Two aphid infestation periods and three cabbage populations were compared, resulting in a total of six pair-wise comparisons with each parasitoid species (see Fig. 1 for an overview of the experimental design). Eight experimental replicates were carried out per combination of cabbage population and aphid-infestation period. After testing the response of the parasitoids to the plants in the Y-tube olfactometer, the number of aphids on dually-infested plants was counted, and the area of leaf tissue consumed by caterpillars was quantified using a transparent plastic sheet with a 1-mm^2^ grid. The bioassays with the two parasitoid species and various pair-wise plant combinations were randomized over the experimental period of three months.

### Headspace Collection

Volatiles were collected from the three herbivore treatments (plants infested with hosts alone, dually infested plants with aphids for seven days plus hosts for the last 24 h, and dually infested plants with aphids for 14 days and hosts for the last 24 h) for each of the two host species and three cabbage populations (18 treatment - population combinations in total, 7–10 replicates each). Plants were grown, inoculated with insects and incubated as described in the *Plant Treatments* section. Volatiles were collected from individual plants using a dynamic headspace collection system in a climate room (21 ± 2 °C, RH 60–70%). The pot containing the plant was wrapped in aluminium foil and the plant was transferred to a clean 35-l glass container. Before sampling, the container was purged for 30 min at 220 ml min^−1^. Compressed air was filtered through activated charcoal before entering the glass container with the plant. Subsequently, volatiles were collected by drawing air from the container through a stainless steel cartridge filled with 200 mg Tenax TA (20/35 mesh; CAMSCO, Houston, TX, USA) for 2 h at a flow of 200 ml min^−1^ using an external pump. Plants of each treatment and cabbage population were randomly selected for volatile trapping on each experimental day. Volatiles were also collected (*n* = 12) from pots containing soil only, which were wrapped in aluminium foil. Volatile compounds detected in these control samples were excluded from the data obtained for the plant samples. The above-ground part of each plant was weighed immediately after volatile trapping. The cartridges filled with Tenax with the trapped headspace samples were dry-purged for 15 min with a nitrogen (N_2_) flow at 50 ml min^−1^ and stored at ambient temperature.

### Chemical Analysis of Volatiles

Separation and identification of plant volatiles was carried out using Thermo Trace Ultra gas chromatography (GC) combined with Thermo Trace DSQ quadrupole mass spectrometer (MS), both from Thermo (Thermo Fisher Scientific, Waltham, USA). The volatiles were thermally released from the Tenax TA adsorbent (Ultra 50:50 thermal desorption unit, Markes, Llantrisant, UK) at 250 °C for 10 min under a helium flow of 20 ml min^−1^, while simultaneously re-collecting the volatiles in a thermally cooled universal solvent trap (Unity, Markes) at 0 °C. Once the desorption process was completed, volatile compounds were released from the cold trap by ballistic heating at 40 °C sec^−1^ to 280 °C and this temperature was maintained for 10 min, while the volatiles being transferred to a ZB-5MSi analytical column with 30 m × 0.25 mm I.D. × 0.25 μm F.T. dimensions and 5 m built-in guard column (Phenomenex, Torrance, CA, USA), in a splitless mode for further separation. The GC oven initial temperature was set to 40 °C and held for 2 min, which was then raised at 6 °C min^−1^ to a final temperature of 280 °C, where it was kept for 4 min under a constant helium flow of 1 ml min^−1^. The DSQ MS was operated in a scan mode with 35–400 amu mass range at 4.70 scans sec^−1^ and spectra were recorded in electron impact ionisation (EI) mode at 70 eV. MS transfer line and ion source were set to 275 and 250 °C, respectively. Tentative identification of compounds was based on comparison of mass spectra with those in the NIST 2005 and Wageningen Mass Spectral Database of Natural Products MS libraries as well as experimentally obtained linear retention indices (LRI).

### Statistics

Of the 1044 tested parasitoids, 93.2% made a choice in the Y-tube olfactometer. The non-responding individuals were excluded from the data analysis. The response variable for the statistical analysis was the proportion of the total number of responding parasitoids choosing dually-infested plants in each experimental replicate. A generalized linear model (GLM) with a binomial distribution and a logit link function was used to compare the proportional preference among the data groups. Parasitoid species (*D. semiclausum* or *M. mediator*), aphid infestation period (7 or 14 days), cabbage population (KIM, OH and WIN), and their interaction terms were entered as fixed factors in the model. If model terms were significant, post-hoc *Tukey-Kramer* multiple comparison tests were performed to reveal differences among the means. For some contrasts specified in the results section, one-sample *t-tests* were used to determine whether the overall distribution of the wasps over the two odor sources deviated from 50:50 (H_0_: μ = 0.5).

The number of aphids counted at 7 or 14 days after inoculation was compared on the three cabbage populations using one-way *ANOVA*. Within populations, leaf damage areas were compared between plants with a single infestation of caterpillars and plants with a dual infestation of both caterpillars and aphids in two-sample t-tests. All univariate statistical tests were performed in SAS 9.3 (SAS Institute Inc., Cary, NC, USA).

The volatile emission patterns, quantified as peak areas of compounds divided by the fresh mass of the plants, were analysed through multivariate data analysis using OPLS-DA (orthogonal projection to latent structures discriminant analysis). The analysis determines if samples belonging to the different treatment groups (singly- and dually-infested plants) can be separated on the basis of quantitative and qualitative differences in their volatile blends. A Y-data matrix of dummy variables assigned a sample to its respective class. The SIMCA-P+ 14.0 software program (UmetricsAB, Umeå, Sweden) then approximates the point ‘swarm’ in X (matrix with volatile compounds) and Y in components in such a way that maximum covariation between the components in X and Y is achieved (Eriksson et al. 2013). To separate predictive from the non-predictive (orthogonal) variation, OPLS-DA further decomposes the X matrix into blocks of structured variation correlated with and orthogonal to Y using the information in the Y matrix (Bylesjö et al. 2006). Data were log-transformed, mean-centred and scaled to unit variance before they were subjected to the analysis. In the analyses, pair-wise comparisons were conducted of the volatile blends of plants with host caterpillars (either *P. xylostella* or *M. brassicae*) alone and dually-infested plants for each cabbage population separately. The parameter Q^2^ in SIMCA is commonly used in the validation of OPLS-DA models; it indicates the predictive ability of the method, and Q^2^ > 0.5 is an indicator for good predictability (Eriksson et al. 2013). Therefore, when the Q^2^ value is larger than 0.5 and at least one significant principal component (PC) was detected, the model is considered to be significant in the discriminant analyses. Per plant population-caterpillar species combination, we also compared the number of compounds emitted by the dually infested plants of which the emission levels increased and decreased relative to the levels emitted by plant infested with caterpillar alone using *χ* ^*2*^-tests.

In addition to a multivariate approach, relative changes in volatile emissions were compared and statistically analysed using univariate statistics. For each compound, the relative emission rate was calculated as the difference between the average amount emitted by plants infested with aphids plus caterpillars and the average amount emitted by plants infested by caterpillars alone divided by the average amount emitted by plants infested by caterpillars alone. Values larger than zero indicate higher emission rates in dually infested plants, whereas values smaller than zero indicate higher emission rates in plants infested with caterpillars alone. To test whether the average relative emission of all compounds together deviated from 0, we used one-sample *t-tests* and to test whether these values differed for the two different aphid infestation periods, we used a two-sample *t-test*.

Total peak areas of all volatile compounds combined were compared using *ANOVA* with plant population and aphid-infestation as fixed factors. Peak areas were log-transformed to meet assumptions of normality and homoscedasticity. If model terms were significant, post-hoc *Tukey-Kramer* multiple comparison tests were performed to reveal differences among the means.

## Results

### *Behavioral Preferences of* D. Semiclausum *and* M. Mediator *for Volatiles Emitted by Host-infested Plants*

**Fig. 2:**
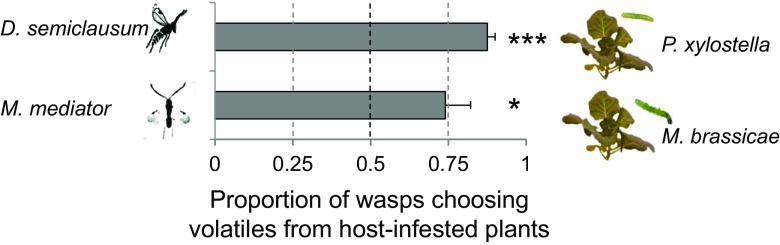
Mean fraction (± SE, *n* = 5) of *Diadegma semiclausum* (*top bar*) and *Microplitis mediator* wasps (*bottom bar*) preferring volatiles from *Brassica oleracea* plants infested with their respective host caterpillars for 24 h when the alternative volatile source was an uninfested plant. Host-infested WIN plants were challenged with 10 *P. xylostella* caterpillars (L2) in the tests with *D. semiclausum* or 30 *M. brassicae* caterpillars (L1) in the tests with *M. mediator*. *Asterisks* (* 0.01 < *P* ≤ 0.05, *** *P* ≤ 0.001) indicate a preference that is significantly different from a 50:50 distribution based on a one-sample *t-test*

When wasps were given the choice between a host-infested and an uninfested plant, both *D. semiclausum* and *M. mediator* significantly preferred volatiles from plants infested by host caterpillars over volatiles from uninfested plants (Fig. 2, *t-test*, *D. semiclausum t*_*4*_ = 15.0, *P* = 0.001; *M. mediator t*_4_ = 2.95, *P* = 0.042). On average 87.5% of the *D. semiclausum* females and 74.0% of *M. mediator* females preferred the volatiles emitted by host-infested plants.

**Fig. 3:**
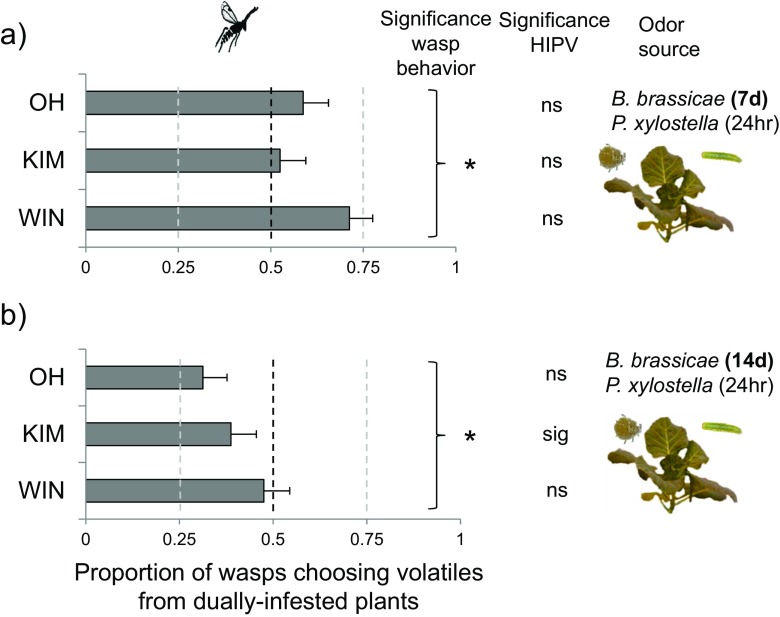
Mean fraction (± SE, *n* = 8) of *Diadegma semiclausum* wasps preferring volatiles emitted by plants infested with hosts (*Plutella xylostella*) and aphids (*Brevicoryne brassicae*) when the alternative volatile source is a plant from the same population infested with only hosts. Three different cabbage (*Brassica oleracea*) populations (OH, KIM, WIN) were compared. Dually-infested plants were challenged with 20 adult aphids for **a** 7 days or **b** 14 days and 10 *P. xylostella* caterpillars (L2) for 24 h, whereas the singly-infested plants were only challenged by 10 *P. xylostella* caterpillars for 24 h. Asterisk indicates whether there is a significant preference for one of the two odor sources using one-sample *t-tests* (μ = 0.5): * 0.01 < *P* ≤ 0.05. The significance levels of pair-wise OPLS-DA models on HIPV compounds emitted by singly- and dually-infested plants are also given in the graph (ns = not significant; sig = significant)

**Fig. 4:**
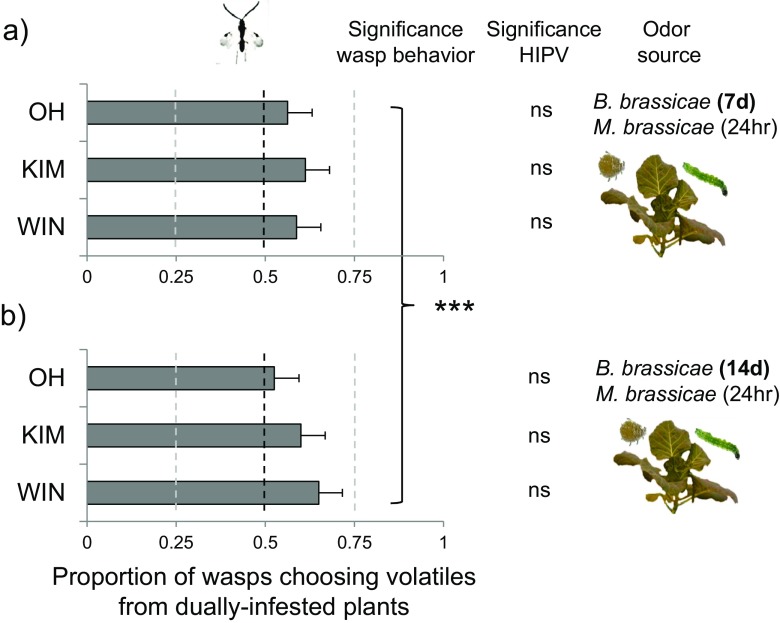
Mean fraction (± SE, *n* = 8) of *Microplitis mediator* preferring volatiles emitted by plants infested with hosts (*Mamestra brassicae*) and aphids (*Brevicoryne brassicae*) when the alternative volatile source is a plant from the same population infested with only hosts. Three different cabbage (*Brassica oleracea*) populations (OH, KIM, WIN) were compared. Dually-infested plants were challenged with 20 adult aphids for **a** 7 days or **b** 14 days and 30 *M. brassicae* caterpillars (L1, one-day-old) for approximately 24 h, whereas the singly-infested plants were only challenged by 30 *M. brassicae* caterpillars for 24 h. Asterisks indicate whether there is a significant preference for one of the two odor sources using one-sample *t-tests* (μ = 0.5): *** *P* ≤ 0.001. The significance levels of pair-wise OPLS-DA models on HIPV compounds emitted by singly- and dually-infested plants are also given in the graph (ns = not significant)

When offered volatiles from plants infested with hosts alone versus hosts plus aphids, the effect of aphid infestation period on proportional preference differed for the two parasitoid species (GLM, parasitoid-infestation period interaction, *χ* ^*2*^_1_ = 7.63, *P* = 0.006). Whereas the preference proportions were similar when comparing the two aphid infestation periods for *M. mediator* (*Tukey-Kramer*; 7 vs. 14 days, *P* = 1.00), they differed for *D. semiclausum* (*P* < 0.001). *Diadegma semiclausum* preferred volatiles from *P. xylostella*-infested plants that were infested with aphids for 7 days over plants infested with only *P. xylostella* (Fig. 3a; *t-test*, *t*_23_ = 2.30, *P* = 0.03). In contrast, when plants had been infested with aphids for 14 days, *D. semiclausum* preferred volatiles emitted by plants that had been infested with only hosts (Fig. 3b; *t-test*; *t*_23_ = 2.66, *P* = 0.014). *Microplitis mediator* was more attracted to volatiles from plants infested with hosts plus aphids than to plants infested with hosts alone at both aphid infestation periods (Fig. 4; *t-test*; *t*_23_ = 26.4, *P* < 0.001). The effect of plant population was not statistically significant (*χ* ^*2*^_2_ = 5.20, *P* = 0.07), neither were the population interaction terms with parasitoid species (*χ* ^*2*^_2_ = 2.09, *P* = 0.35) nor infestation period *χ* ^*2*^_2_ = 0.79, *P* = 0.67) significant. Preference proportions differed marginally when OH and WIN plants were compared (*Tukey-Kramer*, OH vs. WIN, *P* = 0.06, Figs. 3 and 4).

### Aphid Numbers and Caterpillar Leaf Consumption

The mean numbers of aphids did not statistically differ among the three cabbage populations, both on plants infested with aphids for 7 and 14 days for both caterpillar species (One-way *ANOVA* population effect, 7 days of aphid infestation plus *P. xylostella*, *F*_(2, 21)_ = 0.27, *P* = 0.77; 14 days aphid infestation plus *P. xylostella*, *F*_(2, 21)_ = 0.86, *P* = 0.44; 7 days of aphid infestation plus *M. brassicae*, *F*_(2, 21)_ = 1.1, *P* = 0.35; 14 days of aphid infestation plus *M. brassicae*, *F*_(2, 21)_ = 2.7, *P* = 0.094). The overall mean (±SE) number of aphids on plants of the three cabbage populations was 160.5 ± 4.2 after 7 days and 427.1 ± 9.5 after 14 days of aphid infestation.

The amount of leaf damage caused by *P. xylostella* caterpillars did not statistically differ between plants with or without aphids on all populations (two-sample *t-tests*; *t*_6(KIM)_ = 0.33, *P* = 0.75, *t*_8(OH)_ = 0.11, *P* = 0.92, *t*_8(WIN)_ = 0.75, *P* = 0.48). Although overall feeding damage tended to be higher on WIN than on KIM and OH plants, this was not statistically significant (*ANOVA* population effect, *F*_(2, 25_) = 3.39, *P* = 0.051). On average, ten L2 *P. xylostella* caterpillars consumed 27.9 ± 1.5 mm^2^ of leaf tissue in 24 h. There was also no significant difference between the leaf areas consumed by *M. brassicae* caterpillars on plants with or without aphids on all population (*t*_14(KIM)_ = 0.42, *P* = 0.68, *t*_12(OH)_ = 0.09, *P* = 0.93, *t*_14(WIN)_ = 0.35, *P* = 0.73), and the amount of leaf damage did not differ among the three cabbage populations (*ANOVA*, *F*_(2, 43)_ = 2.07, *P* = 0.14). Thirty L1 *M. brassicae* caterpillars consumed on average 81.3 ± 3.1 mm^2^ of leaf tissues in 24 h.

### Volatile Analysis

A total of 51 different volatile compounds were detected in the headspace of the three cabbage populations, across all treatments (Table S1, Online Resource 1). All plants emitted the same compounds but amounts of various compounds varied among treatments (Table S1, Online Resource 1). Pair-wise OPLS-DA of volatiles emitted by plants infested with hosts alone versus dually infested plants showed poor separation of the volatile blends of the two groups for each of the plant populations infested with either caterpillar species. None of the OPLS-DA models were significant after 7 days of aphid infestation and the model was significant only for *P. xylostella*-infested KIM plants after 14 days of aphid infestation (Table S2, Online Resource 1).

**Fig. 5:**
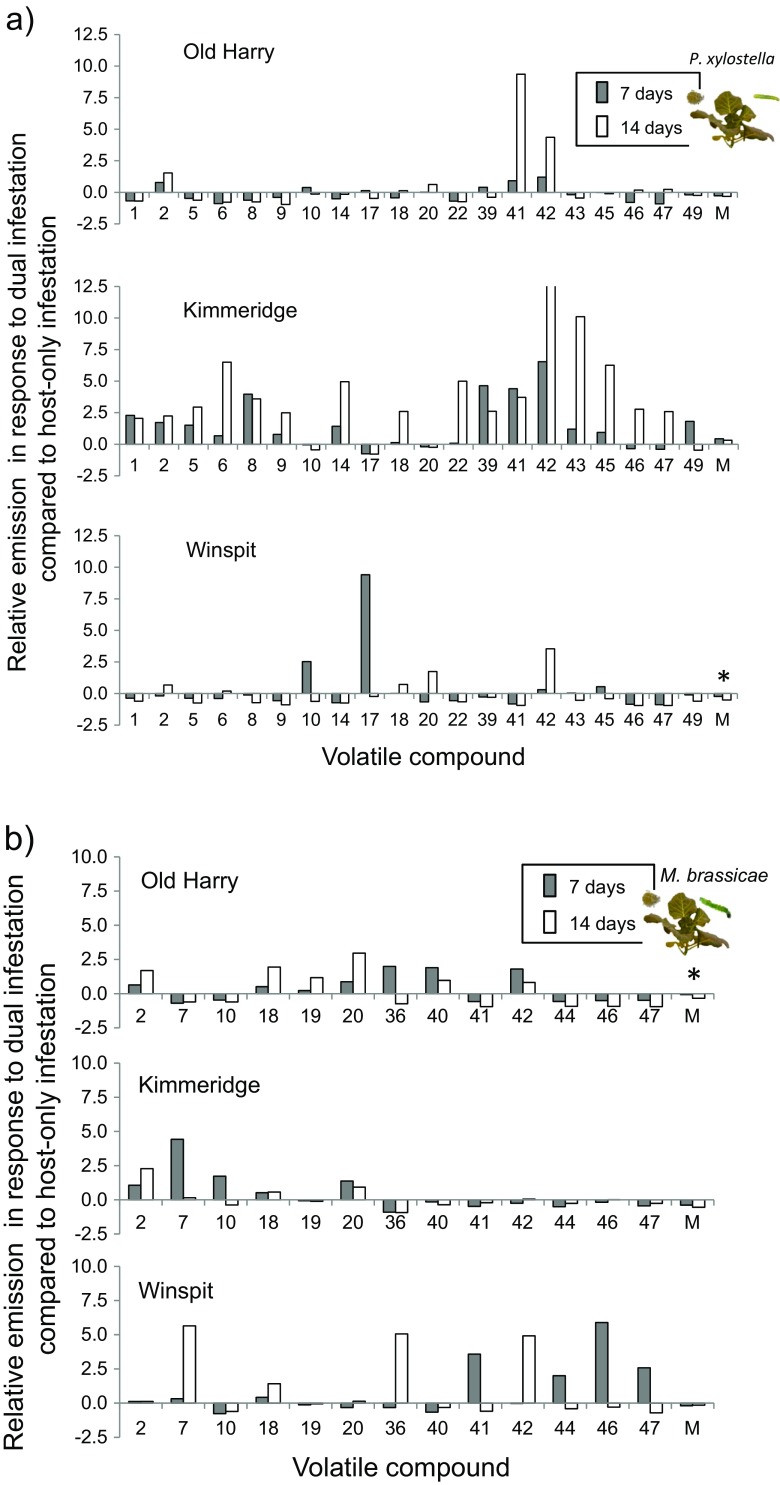
Emission rates of volatiles by plants pre-infested with aphids for 7 (*grey bars*) or 14 days (*white bars*) and caterpillars of **a** *Plutella xylostella* or **b** *Mamestra brassicae* relative to emission rates by plants infested by caterpillars alone for three wild cabbage (*Brassica oleracea*) populations OH, KIM and WIN, respectively. Caterpillars were introduced onto the plant 24 h before the volatiles were collected. Relative emission rates were calculated as the difference between the average amounts emitted by plants infested with aphids plus caterpillars and plants infested by caterpillars alone divided by the amount emitted by plants infested by caterpillars alone. Values larger than zero indicate higher emission rates in dually infested plants, whereas values smaller than zero indicate higher emission rates in plants infested with caterpillars alone. In the graph, only the compounds are shown for which the relative emission rates were higher than 1 or smaller than −1 in at least one of the populations, the mean value for all 51 compounds is given in the last set of bars (M). All M values deviated from zero based on one-sample *t-*tests (*P* < 0.05) and asterisks indicate significant differences (*P* < 0.05) between the M values for the two aphid infestation durations based on two-sample *t-tests*. Numbers refer to the following compounds: 1): (*Z*)-3-hexen-1-ol, 2): 1-octen-3-ol, 4): (*Z*)-3-hexen-1-ol, acetate, 5): hexyl acetate), 6): (*Z*)-3-hexenyl butyrate, 8): (*Z*)-3-hexen-1-ol, 2-methylbutanoate 9): (*Z*)-3-hexen-1-ol, 3-methylbutanoate, 10): linalyl acetate, 11): (*Z*)-4-tert-butylcyclohyxyl acetate, 14): 3-pentanone, 15): 3-methyl-2-pentanone, 17): 3-methylbutanenitrile, 18): dimethyl disulphide, 20): 3-butenyl isothiocyanate, 22) indole, 27): β-myrcene, 33): (*E*)-β-terpineol, 36) (*E*)-DMNT, 39): α-terpineol, 41): β-elemene, 42): bicyclosesquiphellandrene, 43): (*Z,E*)-α-farnesene, 44): β-chamigrene, 45): (*E,E*)-α-farnesene, 46): germacrene A, 47): (*Z*)-α-bisabolene, and 49): 4-ethenyl cyclohexene

On average, volatile emission rates were reduced in plants infested with aphids plus caterpillars compared to plants infested with caterpillars alone for both caterpillar species on all three populations (Fig. 5, last sets of bars), except for KIM plants dually infested with aphids and *P. xylostella* caterpillars which emitted volatiles in larger volumes than the plants infested with *P. xylostella* alone. The duration of aphid-infestation on the relative change in volatile emission was only significant for WIN plants infested with *P. xylostella* and OH plants infested with *M. brassicae*, where in both cases the longer infestation period reduced relative emission of volatiles the most (Fig. 5).

The number of compounds for which the emission decreased or increased also differed among the population-treatment combinations. In OH and WIN plants infested with *P. xylostella* plus aphids, the number of compounds for which the emission decreased compared to caterpillar-only infestation was significantly higher than the number of compounds for which the emission increased, but only when the aphids were on the plants for 14 days (*χ* ^*2*^_1_ = 16.5, *P* < 0.001 for both OH and WIN). KIM plants co-infested with aphids for 7 days, emitted significantly more compounds in higher than in lower rates compared to plants infested with caterpillars alone (*χ* ^*2*^_1_ = 5.67, *P* = 0.02), whereas the number of compounds for which the emission increased and decreased was similar after 14 days of aphid infestation (Table S1A, Online Resource 1). For *M. brassicae*, in each of the three populations, more compounds were emitted at lower than at higher levels relative to the levels emitted by plants infested with *M. brassicae* alone, except for OH plants co-infested with aphids for 7 days where the number of compounds with increased and decreased emission levels was similar (OH: 7 days, χ^*2*^_1_ = 0.02, *P* = 0.89; 14 days, *χ* ^*2*^_1_ = 10.4, *P* = 0.001; KIM: *χ* ^*2*^_1_ = 14.3, *P* < 0.001; 14 days, *χ* ^*2*^_1_ = 26.8, *P* < 0.001, WIN, 7 and 14 days, *χ* ^*2*^_1_ = 12.3, *P* < 0.001, Table S1B, Online Resource).

**Fig. 6:**
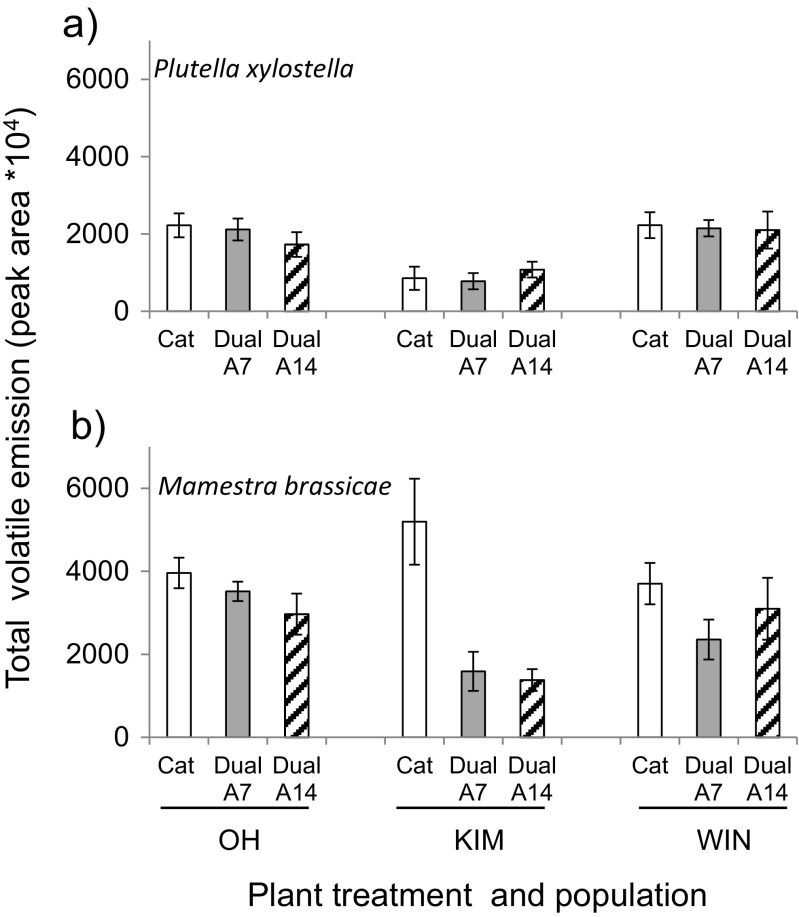
Total volatile emission by *Brassica oleracea* plants originating from three wild cabbage populations (OH, KIM and WIN) infested with only caterpillars for 24 h (*white bars*), pre-infested with aphids (*Brevicoryne brassicae*) for 7 days before introduction of the caterpillars for the last 24 h (grey bars) or 14 days and caterpillars for the last 24 h (*dashed bars*). Plants were either infested with caterpillars of **a** *Plutella xylostella* or **b** *Mamestra brassicae*. Bars represent the mean ± SE peak area in chromatograms corrected by fresh plant weight (sample sizes ranged between 7 and 10)

There were substantial differences in the total volatile production among populations and in response to the two herbivores (Fig. 6). It was higher in plants exposed to *M. brassicae* than in plants exposed to *P. xylostella.* Moreover, in plants exposed to *P. xylostella*, the effect of prior aphid infestation on total volatile production was not significant (*F*_2,62_ = 0.28, *P* = 0.75) and there was only an effect of plant population (*F*_2,62_ = 13.8, *P* < 0.001) with KIM plants producing lower overall amounts of volatiles than the other two populations. In plants exposed to *M. brassicae* there was both an effect of plant treatment (*F*_2,63_ = 9.47, *P* < 0.001) and plant population (*F*_2,63_ = 5.92, *P* = 0.004). In line with the previous analyses, total volatile production was reduced in plants pre-infested with aphids, but did not differ in relation to aphid-infestation duration (*Tukey-Kramer*, *M. brassicae* alone vs. dual both time periods: *P* < 0.01). The only compound that was systematically emitted in higher concentration in plants with aphids and *P. xylostella* caterpillars in all three populations was bicyclosesquiphellandrene (tentatively identified) (Fig. 5a). Such a consistency was not found for plants infested with *M. brassicae* (Fig. 5b).

## Discussion

Our data show that the relative attraction of parasitoids to volatiles emitted by plants dually infested with hosts and aphids and those emitted by plants infested with hosts alone is species-specific and also depends on the duration of pre-infestation with aphids. After 7 days of aphid feeding both parasitoid species were more attracted to HIPVs emitted by dually-infested plants than to those emitted by plants damaged by their respective hosts alone. When aphid feeding was extended to 14 days, dually infested plants were still preferred by *M. mediator,* but *D. semiclausum* now preferred volatiles from plants with only hosts. Cabbage plant population did not affect these behavioral choices. Chemically, there were differences among the plant populations in their response to treatments with only caterpillars or a combination of aphids and caterpillars. In response to dual infestation with *M. brassicae* caterpillars and aphids, emission rates in dually-infested plants were reduced compared to emission rates in plants infested with only hosts. This was the case for all three plant populations and the two aphid infestation periods. However, for *P. xylostella* this pattern was only recorded in OH and WIN plants when aphid infestation was extended. In KIM plants more compounds were produced at higher than at lower levels when infested with caterpillars (*P. xylostella*) and aphids for 7 days.

Extended aphid infestation resulted in a higher aphid density and this may have affected plant volatile biosynthesis and attractiveness of the volatiles to the parasitoids. Co-infestation by non-host aphids may enhance or attenuate the attraction of a parasitoid depending on the length of the aphid infestation period and possibly aphid density. The parasitoid *Cotesia glomerata* also switched its preference for plants dually-infested with hosts (*Pieris brassicae* caterpillars) and *B. brassicae* aphids at low aphid densities to preference for host-only infested plants at high aphid densities (Ponzio et al. 2016). Other studies have investigated the effect of simultaneous feeding by host and non-host herbivores on HIPV induction and the response of natural enemies (Dicke et al. 2009; Erb et al. 2010; Zhang et al. 2013; Zhang et al. 2009), but only few of these studies have addressed the effect of non-host infestation period. For instance, infestation with non-prey whiteflies reduced the attraction of carnivorous mites to plants infested with prey spider mites and the degree of interference increased with whitefly density (Zhang et al. 2009). *Microplitis mediator* was consistently more attracted to plants dually infested with aphids and hosts over plants infested with hosts alone, regardless of the aphid infestation period. The contrasting preferences of *D. semiclausum* and *M. mediator* at 14 days of aphid infestation suggest that the effect of aphid-density or aphid-infestation duration on volatile-mediated foraging is host-parasitoid interaction specific.

To maximize reproductive success and extend longevity, parasitic wasps need resources, mainly sugar-rich resources such as floral nectar (Amat et al. 2012; Geneau et al. 2012; Harvey et al. 2012). Aphid honeydew can serve as a food source for the parasitoids when nectar is scarce (Faria et al. 2008). *Aphytis melinus* parasitoids commonly feed on honeydew from non-host hemipteran species in the field and more than 50% of the field-collected female parasitoids of this species had fed on honeydew (Tena et al. 2013). The availability of an alternative food source, such as aphid honeydew on dually-infested plants could increase the plant’s value to parasitoids (Stapel et al. 1997), which may also explain why wasps preferred the HIPV from plants infested with host caterpillars plus non-host aphids. Whether products of the aphids themselves enhance the attractiveness of the volatile blend emitted by plants infested with host and aphids remains to be investigated. Aphid co-infestation seems to affect the behavior of *M. mediator* more consistently than that of *D. semiclausum*. The response of parasitoids to food-related stimuli, such as flower color and odor depends on the hunger state of the individual. Food-deprived wasps prefer odors related to food over those related to hosts (Wäckers 1994). In our study, the wasps were provided with fresh honey and sugar water daily, but their hunger status was not assessed before the experiments. To what extent aphid honeydew may play a role in the attractiveness of dually infested plants to wasps remains to be determined.

Host-selection behavior by parasitoids attacking herbivorous hosts consists of different phases of which habitat and host pant location are considered the first steps and these are often mediated by HIPV (Vet and Dicke 1992; Vinson 1998). Once a host-infested plant has been found, parasitism success depends on the ability to find the hosts on that plant, to parasitize the hosts and for the parasitoid offspring to successfully develop inside or on these hosts (Vet and Dicke 1992; Vinson 1998). A previous study on host quality mediated by the host plant using the same tritrophic systems as in this study reported that development of *P. xylostella* and *D. semiclausum*, but not that of *M. brassicae* and *M. mediator* was positively affected by aphid presence (Li et al. 2014). Body mass of *P. xylostella* and, thus, the amount of biomass available for its parasitoids, was positively affected by a low density of co-infesting *B. brassicae* aphids and negatively by a high density of co-infested aphids (Kroes et al. 2015). These results suggests that the direction of aphid-induced effects on both the performance and behavior of parasitoids can be aphid-density dependent. To determine whether successful location of host-infested plants increases successful parasitism and ultimately leads to higher reproductive success needs to be assessed for the two parasitoids investigated here considering the other steps that ultimately determine parasitism success (see e.g. Soler et al. 2007; Wei et al. 2011). Interestingly, early infestation with *B. brassicae* in a common garden experiment using the same three cabbage populations as in this study did not affect parasitism of *P. xylostella* or parasitoid-host dynamics during the growing season (Li et al. 2016b). This suggest that under field conditions the effect of aphid presence or their density on parasitoid foraging behavior may not be very strong.

We showed that aphid feeding on host-infested plants may benefit parasitoids by increased detectability of the HIPV-blend compared to the blend emitted by plants damaged by caterpillars only, but also that the efficiency of a parasitoid in finding hosts can be compromized when other herbivore species are feeding on the same plant (see also De Rijk et al. 2013). The effect of aphid infestation on the emission rates of plant volatiles was mostly negative. The effects of aphid infestation in reducing volatile emission rates were most pronounced when plants were infested with *M. brassicae*, irrespective of the duration of the aphid infestation. In plants infested with *P. xylostella* reduced emission rates were only recorded in OH and WIN plants after extended aphid feeding. Reduced emission in plants pre-infested with aphids before the host caterpillars are introduced provides evidence suggesting the involvement of SA-JA antagonism (Thaler et al. 2012; Zhang et al. 2009). In a recent study (Li et al. 2016a), no evidence was found for JA-SA antagonism in wild cabbage plants based on expression of a JA- and a SA-regulated marker gene. This result was not affected by the order of introduction of the chewing herbivore (*P. xylostella*) and the phloem feeding aphid (*B. brassicae*), or herbivore species identity (*P. xylostella*, *M. brassicae or P. brassicae*).

Reduced volatile emission could also be the result of reduced feeding by the caterpillars when aphids were present on the plant, either through direct interaction with the aphids or through reduced quality of the host plant in response to prolonged aphid feeding. However, feeding damage inflicted by the caterpillars was similar on plants pre-infested with aphids and plants free of aphids, suggesting that the effect of reduced feeding damage is minimal or very subtle. Most importantly, the negative interaction between aphids and caterpillars, in terms of reduced volatile emission, did not result in reduced attraction of *M. mediator.* Linking parasitoid behavior and chemistry for the *P. xylostella-D. semiclausum* interaction was even more difficult because, here, there were also plant-population specific responses in terms of volatile emission rates (OH and WIN vs KIM). Aphid interference with HIPV induction was shown in OH and WIN when aphid infestation was extended and also negatively affected *D. semiclausum* supporting a role for JA-SA antagonism in only two of the plant populations, if this is indeed the underlying mechanism.

Enhanced attraction to *P. xylostella*-damaged plants co-infested with aphids for 7 days could not be explained by the data on headspace volatiles. However, reduced attraction to dually-infested plants when aphid infestation was extended to 14 days could be explained by a critical reduction of key components in the HIPV blend that are important cues for the attraction of *D. semiclausum* or by the increased emission of the tentatively identified terpene bicyclosesquiphellandrene, the only compound that systematically increased in plants exposed to aphids for 14 days and co-infested with *P. xylostella*. The effect of this compound in reducing the attractiveness of the blend was not further investigated. The two parasitoid species clearly differ in the specific blend characteristics that they use during foraging. This may result from differences in the sensitivity to specific compounds, the range of compounds that triggers a sensory response, and the processing of stimuli in the brain and is likely to be determined by the ecology and genetic constraints of the parasitoid species. Rearing history may also play a role in the ability to detect more subtle differences between HIPV blends (Gols et al. 2012). However, *Cotesia rubecula* still preferred host-induced volatiles emitted by wild cabbage plants over those emitted by a cabbage cultivar on which the parasitoid had been reared for many generations (Gols et al. 2011). Dietary specialisation in *D. semiclausum* may further explain the more pronounced sensitivity to subtle changes in the HIPV blend in this species (Vet and Dicke 1992; Steidle and van Loon 2003). However, in a study with *D. semiclausum* and the generalist congeneric parasitoid *D. fenestrale* the ability to discriminate between host and non-host-infested plants was similar for the two parasitoid species (Gols et al. 2012). These results suggest that phylogenetic constraints also determine responses to HIPVs.

Genotypes and cultivars of the same species can vary in HIPV blends emitted in response to an individual herbivore species (Köllner et al. 2008; Lou et al. 2006; Poelman et al. 2009). A previous study has demonstrated that plants from the same three wild cabbage populations as used in this study vary in their HIPV profiles in response to infestation by *Pieris rapae* caterpillars (Gols et al. 2011). Here, we investigated dual infestation and found that the preference patterns of the parasitoids for volatiles from host-infested plants and plants infested by hosts plus non-host aphids were similar across the three wild cabbage populations. Despite the differences in HIPV emissions among the populations, the response of the parasitoids to the herbivore treatments appeared to be resilient to this variation, at least when treatments within populations are compared.

To summarize, our data show that pre-infestation with phloem-feeding aphids before introducing tissue-feeding caterpillars onto the plants generally reduced the emission of many volatile compounds in the HIPV blend of wild cabbage suggesting that SA-JA antagonism is involved (Zhang et al. 2009). This reduced volatile emission does not necessarily have a negative effect on parasitoid attraction. Whether this is the case depends on the specific host-parasitoid interaction in combination with the duration of pre-infestation with aphids. Our data further show that it is important to include several strains or populations of a plant species. If only plants from the KIM population had been included, for which the plant’s chemical response deviated from the other two populations, linking parasitoid behavior to plant volatile chemistry would have been even more difficult. This study further illustrates that the relationship between parasitoid attraction and characteristics of the HIPV blend is extremely complex (see also Ponzio et al. 2016; Webster et al. 2010). Complementary effects of compounds, synergistic and antagonistic interactions between blend components, as well as differences in sensory sensitivity to individual compounds may explain why it is so difficult to elucidate what makes an HIPV blend attractive to a parasitoid.

## Electronic supplementary material

ESM 1 (DOCX 59 KB)
