# Supplementary material for: Does Aphid Infestation Interfere with Indirect Plant Defense against Lepidopteran Caterpillars in Wild Cabbage?
Source: J Chem Ecol. 2017 Apr 12;43(5):493–505. doi: 10.1007/s10886-017-0842-z (PMC5487765; doi:10.1007/s10886-017-0842-z)
Supplement: Supplementary file 1 — (DOCX 59 KB) [file 10886_2017_842_MOESM1_ESM.docx]

Does aphid infestation interfere with indirect plant defence against lepidopteran caterpillars in wild cabbage?

Journal of Chemical Ecology

Yehua Li, Berhane T. Weldegergis, Surachet Chamontri, Marcel Dicke and Rieta Gols*

*Laboratory of Entomology, Wageningen University, P.O. Box 16, 6700 AA Wageningen, the Netherlands*

^*^ Correspondence author: [rieta.gols@wur.nl](mailto:rieta.gols@wur.nl),

**Table 1** Volatile emissions by plants of three wild cabbage populations (*Brassica oleracea*), OH, KIM, and WIN, in response to feeding by two caterpillar species (a) *Plutella xylostella* (Px) or (b) *Mamestra brassicae* (Mb) that were either feeding alone or together with aphids (*Brevicoryne brassicae*) for 7 or 14 days (Bb(7d) and Bb(14d)).

| a) |  |  |  |  |  |  |  |  |  |
| --- | --- | --- | --- | --- | --- | --- | --- | --- | --- |
| Compound |  | OH |  |  | KIM |  |  | WIN |  |
|  | Px | Bb(7d)+Px | Bb(14d)+Px | Px | Bb(7d)+Px | Bb(14d)+Px | Px | Bb(7d)+Px | Bb(14d)+Px |
| **Alcohols** |  |  |  |  |  |  |  |  |  |
| ^1^(*Z*)-3-Hexen-1-ol (928-96-1)^#^ | 4.5±2.5^!^ | **1.4±0.3** | **1.4±0.3** | 2.3±1.2 | 7.6±6.5 | **7.1±2.9** | 4.5±1.9 | 2.8±1.4 | **1.7±1.0** |
| ^2^1-Octen-3-ol (3391-86-4) | 6.6±1.2 | 11.8±3.1 | **16.8±2.9** | 5.0±0.6 | 13.7±5.0 | **16±4.0** | 10.6±2.0 | 8.5±1.4 | 18.0±3.5 |
| **Esters** |  |  |  |  |  |  |  |  |  |
| ^3^(*Z*)-2-Penten-1-ol, acetate  (42125-10-0) | 1.5±0.4 | **0.80±0.16** | **0.7±0.2** | 2.1±1.6 | 3.7±3.0 | 2.3±1.1 | 2.3±1.2 | 0.9±0.2 | 0.31±0.10 |
| ^4^(*Z*)-3-Hexen-1-ol, acetate (3681-71-8) | 373±186 | **159±51** | **100±20** | 252±183 | 474±385 | **463±207** | 248±92 | 167±51 | **88±40** |
| ^5^Hexyl acetate (142-92-7) | 3.5±1.3 | **1.8±0.8** | **1.3±0.3** | 2.0±0.8 | 5.0±3.8 | **7.8±3.6** | 2.4±0.6 | 1.5±0.4 | **0.60±0.16** |
| ^6^(*Z*)-3-Hexenyl butyrate  (16491-36-4) | 0.8±0.7 | **0.08±0.03** | **0.17±0.06** | 0.10±0.05 | 0.16±0.11 | **0.7±0.4** | 0.6±0.3 | 0.35±0.15 | 0.7±0.4 |
| ^7^Methyl salicylate (119-36-8) | 4.3±3.1 | **7.1±2.9** | 4.1±1.2 | 5.1±3.8 | 2.6±0.9 | 0.73±0.12 | 2.7±1.6 | 3.9±1.8 | **1.1±0.2** |
| ^8^(*Z*)-3-Hexen-1-ol,  2-methylbutanoate (53398-85-9) | 1.3±0.73 | **0.47±0.15** | **0.30±0.11** | 0.30±0.19 | 1.5±1.2 | **1.4±0.7** | 0.7±0.2 | 0.6±0.3 | **0.18±0.10** |
| ^9^(*Z*)-3-Hexen-1-ol,  3-methylbutanoate (?) | 0.29±0.19 | **0.17±0.09** | **0.01±0.01** | 0.05±0.03 | 0.08±0.08 | **0.16±0.12** | 0.11±0.08 | 0.05±0.04 | 0.01±0.01 |
| ^10^Linalyl acetate | 0.8±0.3 | **1.1±0.3** | 0.67±0.14 | 2.6±1.7 | 2.5±1.7 | 1.4±0.5 | 1.1±0.4 | 4.0±3.0 | **0.42±0.13** |
| ^11^(*Z*)-4-tert-Butylcyclo-  hexyl acetate (32210-23-4) | 1.9±0.5 | 1.7±0.5 | 1.5±0.3 | 3.8±1.9 | 2.8±1.6 | **1.7±0.6** | 2.4±0.4 | 2.7±1.4 | 1.4±0.4 |
| ^12^α-Terpinyl acetate (80-26-2) | 0.18±0.06 | 0.21±0.06 | 0.11±0.03 | 0.37±0.15 | 0.26±0.08 | 0.21±0.11 | 0.21±0.05 | 0.23±0.10 | 0.11±0.03 |
| **Ketones** |  |  |  |  |  |  |  |  |  |
| ^13^3-Methyl-2-butanone (563-80-4) | 1.1±0.8 | **0.13±0.11** | **0.05±0.04** | 1.5±0.5 | 2.4±0.9 | **0.35±0.14** | 1.8±0.7 | 0.7±0.4 | **0.6±0.3** |
| ^14^3-Pentanone (96-22-0) | 1.9±0.4 | **0.92±0.14** | 1.6±0.5 | 1.4±0.5 | 3.3±2.5 | **8.1±5.6** | 6.3±4.3 | 1.7±0.4 | 1.6±0.4 |
| ^15^3-Methyl-2-pentanone  (565­-61-7) | 6.4±3.5 | **1.7±0.9** | **0.41±0.08** | 3.9±1.1 | 3.5±1.2 | **1.4±0.5** | 7.4±4.4 | 2.8±0.8 | **0.81±0.19** |
| ^16^α-Isomethylionone (127-51-5) | 0.40±0.17 | 1.4±0.7 | 0.45±0.11 | 1.5±0.8 | 1.2±0.6 | **0.6±0.2** | 1.5±0.7 | 1.1±0.6 | 0.28±0.05 |
| **S- & N- containing compounds** |  |  |  |  |  |  |  |  |  |
| ^17^3-Methylbutanenitrile  (625-28-5) | 1.8±0.6 | 2.0±0.8 | 0.9±0.2 | 2.4±1.5 | 0.6±0.3 | 0.51±0.13 | 0.3±0.2 | 3.4±2.4 | 0.25±0.08 |
| ^18^Dimethyl disulphide (624-92-0) | 7.6±2.2 | **4.3±0.8** | 8.5±1.5 | 13.9±4.5 | 16±6.2 | **50±19** | 7.1±1.5 | 7.1±2.6 | 12.2±2.3 |
| ^19^Dimethyl trisulfide (3658-80-8) | 2.9±1.1 | 1.7±0.3 | 2.5±0.5 | 6.0±2.5 | 6.2±2.7 | 10.1±2.2 | 4.0±1.3 | 2.3±0.5 | 2.4±0.4 |
| ^20^3-Butenyl isothiocyanate  (3386-97-8) | 27.4±9.3 | 28±7.1 | 44±13 | 13.7±5.5 | 11±4.5 | 10.4±3.5 | 58±15 | 19.6±2.4 | **159±45** |
| ^21^Benzyl nitrile (140-29-4) | 7.3±3.7 | **10.5±2.6** | 3.4±1.0 | 0.41±0.18 | 0.32±0.08 | 0.38±0.09 | 1.4±0.4 | 1.4±0.4 | 1.1±0.3 |
| ^22^Indole (120-72-9) | 5.1±2.0 | **1.5±0.6** | **1.3±0.5** | 0.9±0.8 | 1.0±0.4 | 5.6±3.5 | 4.8±3.1 | 2.0±1.0 | **1.7±0.7** |
| **Terpenoids** |  |  |  |  |  |  |  |  |  |
| ^23^α-Thujene (3917-48-4) | 273±32 | 294±44 | 223±47 | 69±44 | 135±56 | 60±29 | 284±57 | 302±33 | 298±78 |
| ^24^α-Pinene (80-56-8) | 81.9±9.2 | 82±14 | 68±11 | 29±14 | 41±16 | 25±10 | 93±18 | 84.1±8.2 | **77±18** |
| ^25^Sabinene (3387-41-5) | 661±75 | 758±117 | **587±100** | 195±121 | 364±151 | 163±78 | 703±143 | 750±87 | 701±174 |
| ^26^β-Pinene (127-91-3) | 66.1±6.6 | 64.1±9.8 | 53.7±9.0 | 19±10 | 28±11 | 21.0±9.4 | 65±13 | 67.8±7.2 | 58±14 |
| ^27^β-Myrcene (123-35-3) | 198±23 | 213±32 | 179±31 | 59±31 | 104±43 | 49±22 | 216±42 | 227±26 | 218±56 |
| ^28^α-Terpinene (99-86-5) | 25.0±6.4 | **20.5±3.3** | 24.5±7.7 | 5.8±3.3 | 11.4±6.0 | 4.7±2.3 | 25.9±6.7 | 27.3±5.9 | 27.1±9.8 |
| ^29^Limonene (138-86-3) | 272±31 | 299±42 | 262±55 | 102±46 | 182±73 | 97±41 | 326±66 | 330±35 | **306±71** |
| ^30^1,8-Cineole (470-82-6) | 68.9±8.3 | 76±13 | 60±11 | 19±11 | 28±13 | 16.2±7.5 | 65±14 | 46.7±9.9 | 66±19 |
| ^31^(*E*)-β-Ocimene (13877-91-3) | 5.8±1.0 | 5.5±1.0 | 4.8±0.9 | 2.3±1.5 | 3.1±1.3 | 1.9±0.7 | 6.2±1.4 | 6.4±0.7 | 5.3±1.3 |
| ^32^γ-Terpinene (99-85-4) | 17.2±4.4 | 15±2.4 | 16.7±5.1 | 4.2±2.2 | 8.0±4.1 | 3.5±1.6 | 17.8±4.6 | 19.3±4.1 | 19±6.7 |
| ^33^(*E*)-β-Terpineol (138-87-4) | 2.4±0.3 | 2.8±0.5 | **1.5±0.3** | 0.6±0.4 | 1.2±0.5 | 0.7±0.3 | 2.6±0.6 | 2.4±0.4 | 2.4±0.7 |
| ^34^α-Terpinolene (586-62-9) | 11.7±2.5 | 10.1±1.8 | 12±2.8 | 3.1±1.7 | 5.5±2.7 | 2.6±1.1 | 13.3±3.0 | 12.5±2.4 | 12.3±4.1 |
| ^35^Linalool (78-70-6) | 1.8±0.5 | **2.1±0.3** | 1.3±0.3 | 6.1±5.2 | 2.2±1.0 | 3.2±1.8 | 3.1±1.6 | 5.7±2.9 | 2.9±0.8 |
| ^36^(*E*)-DMNT^#^ (19945-61-0) | 48±20 | 8.9±1.9 | 15.3±7.4 | 6.1±2.0 | 11.0±3.8 | 5.3±1.9 | 2.6±0.7 | 2.7±1.3 | **0.68±0.11** |
| ^37^Menthol (89-78-1) | 0.51±0.18 | **0.40±0.07** | 0.5±0.3 | 2.9±2.5 | 1.8±1.3 | 0.5±0.2 | 1.0±0.5 | 1.6±1.2 | 0.17±0.07 |
| ^38^4-Terpineol (562-74-3) | 0.57±0.06 | 0.68±0.11 | 0.59±0.13 | 0.18±0.08 | 0.38±0.12 | 0.17±0.08 | 0.7±0.2 | 0.66±0.18 | 0.9±0.2 |
| ^39^α-Terpineol (98-55-5) | 2.9±1.1 | **4.0±0.8** | 1.7±0.6 | 0.39±0.19 | 2.2±1.1 | **1.4±1.0** | 6.4±2.6 | 4.7±1.9 | 4.5±2.2 |
| ^40^β-Cubebene (13744-15-5) | 0.21±0.05 | **0.10±0.03** | 0.12±0.06 | 0.16±0.04 | 0.13±0.06 | 0.10±0.04 | 0.3±0.2 | 0.12±0.04 | **0.07±0.02** |
| ^41^β-Elemene (33880-83-0) | 0.46±0.43 | 0.9±0.6 | **4.8±2.3** | 1.3±0.9 | 7.2±5.7 | **6.3±3.1** | 8.6±4.3 | 1.5±0.8 | **0.5±0.3** |
| ^42^Bicyclosesquiphellandrene  (54324-03-7) | 0.05±0.02 | 0.12±0.05 | **0.28±0.07** | 0.02±0.02 | 0.14±0.05 | **0.40±0.15** | 0.08±0.04 | 0.10±0.04 | 0.35±0.19 |
| ^43^(*Z*,*E*)-α-Farnesene (26560-14-5) | 0.8±0.3 | 0.65±0.18 | **0.41±0.12** | 0.08±0.03 | 0.16±0.07 | **0.8±0.2** | 0.5±0.2 | 0.5±0.2 | 0.20±0.07 |
| ^44^β-Chamigrene (15401-86-2) | 0.88±0.67 | **0.33±0.09** | 1.2±0.5 | 0.47±0.09 | 0.29±0.08 | **0.9±0.4** | 1.7±0.6 | 0.40±0.11 | 0.25±0.08 |
| ^45^ (*E*,*E*)-α-Farnesene  (502-61-4) | 18.6±6.7 | 18.4±5.7 | 15.7±5.8 | 2.7±0.9 | 5.2±1.6 | **19±9.2** | 9.2±2.3 | 14.1±6.3 | 5.4±1.1 |
| ^46^Germacrene A (28387-44-2) | 0.7±0.7 | **0.15±0.08** | 0.9±0.4 | 0.24±0.14 | 0.16±0.06 | **0.9±0.5** | 1.2±0.6 | 0.18±0.08 | 0.06±0.05 |
| ^47^(*Z*)-α-Bisabolene (29837-07-8) | 0.22±0.2 | **0.02±0.02** | 0.28±0.14 | 0.07±0.04 | 0.04±0.02 | **0.25±0.15** | 0.4±0.2 | 0.05±0.03 | 0.02±0.01 |
| ^48^Nerolidol (7212-44-4) | 0.45±0.13 | 0.38±0.10 | 0.29±0.07 | 0.30±0.13 | 0.6±0.4 | 0.34±0.11 | 0.21±0.07 | 0.22±0.09 | **0.12±0.06** |
| **other or unknown** |  |  |  |  |  |  |  |  |  |
| ^49^4-Ethenyl cyclohexene  (100-40-3) | 3.4±2.4 | 2.7±1.8 | 2.6±1.1 | 1.9±1.1 | 5.2±3.3 | 1.0±0.3 | 2.8±1.4 | 2.5±1.8 | 1.1±0.6 |
| ^50^Unknown | 0.8±0.2 | 0.69±0.18 | 0.68±0.18 | 0.7±0.3 | 0.6±0.2 | 0.8±0.2 | 1.0±0.3 | 0.60±0.11 | **0.63±0.14** |
| ^51^Unknown | 1.6±0.4 | 1.8±0.4 | **1.2±0.2** | 1.8±0.6 | 1.7±0.5 | 1.7±0.3 | 2.1±0.5 | 1.4±0.4 | **1.3±0.3** |
| Number of compounds that  increased in dually infested plants^a^ | | 21 | 11 |  | 34^*^ | 24 |  | 21 | 11 |
| Number of compounds that  decreased in dually infested plants^b^ | | 30 | 40^***^ |  | 17 | 27 |  | 30 | 40^***^ |

^!^Volatile emissions are given as mean chromatogram peak area ± SE (n=7-10) per g fresh weight of foliage divided by 10^4^.

^#^CAS numbers of the compounds are given in brackets after the compound names.

^@^Numbers in superscript before the compound names serve as identifiers for the compounds in Fig. 4

^a^Numbers of compounds of which the emission was higher in dually-infested plants than in plants infested with caterpillars alone.

^b^Numbers of compounds of which the emission was lower in dually-infested plants than in plants infested with caterpillars alone.

Asterisks indicate significant differences between the number of compounds that increased and decreased based on χ^2^-test, * P<0.05, *** P<0.001.

^#^ (*E*)-4,8-dimethyl-nona-1,3,7-triene

| b) |  | |  |  |  |  |  |  |  |  |
| --- | --- | --- | --- | --- | --- | --- | --- | --- | --- | --- |
| Compound |  | | OH |  |  | KIM |  |  | WIN |  |
|  | Mb | | Bb(7d)+Mb | Bb(14d)+Mb | Mb | Bb(7d)+Mb | Bb(14d)+Mb | Mb | Bb(7d)+Mb | Bb(14d)+Mb |
| **Alcohols** |  |  | |  |  |  |  |  |  |  |
| ^1@^(*Z*)-3-Hexen-1-ol | 34±6.2 | | **17.2±2.8** | **18.9±4.6** | 56±14 | **17.6±5.7** | **16.6±4.0** | 12.4±3.5 | 8.3±1.9 | 9.3±4.7 |
| ^2^1-Octen-3-ol | 7.6±1.3 | | **12.4±2.2** | **20.3±4.9** | 5.4±1.6 | **11.1±1.9** | **17.7±2.7** | 9.2±1.9 | 10.3±2.8 | 10.4±1.8 |
| **Esters** |  | |  |  |  |  |  |  |  |  |
| ^3^ (*Z*)-2-Penten-1-ol, acetate | **6.2±1.5** | | **5.1±0.8** | **2.4±0.3** | 33±13 | **3.6±0.9** | **3.1±0.7** | 2.4±0.5 | 1.9±0.4 | **1.1±0.3** |
| ^4^ (*Z*)-3-Hexen-1-ol, acetate | 1832±400 | | **1179±195** | **869±165** | 3598±811 | **724±158** | **647±156** | 631±140 | **353±66** | **280±120** |
| ^5^ Hexyl acetate | 9.1±2.3 | | **5.1±1.0** | **6.6±1.5** | 33±13 | **3.4±0.8** | **6.0±3.0** | 3.1±0.9 | 2.0±0.5 | **1.4±0.6** |
| ^6^ (*Z*)-3-Hexenyl butyrate | 1.9±0.5 | | 1.3±0.4 | 1.0±0.3 | 7.7±3.2 | **1.4±0.7** | **0.8±0.3** | 2.4±0.7 | **0.9±0.3** | 1.7±1.1 |
| ^7^Methyl salicylate | 29±19 | | **8.8±1.5** | 11.5±7.6 | 1.6±0.6 | **8.7±4.4** | 1.9±0.8 | 3.7±1.4 | 4.8±2.6 | **24±12** |
| ^8^ (*Z*)-3-Hexen-1-ol,  2-methylbutanoate | 3.1±0.8 | | 2.4±0.6 | **2.0±0.3** | 11.0±3.8 | **0.9±0.2** | **1.2±0.3** | 1.3±0.4 | **0.68±0.13** | **0.9±0.4** |
| ^9^ (*Z*)-3-Hexen-1-ol,  3-methylbutanoate | 1.8±0.5 | | 1.6±0.3 | 1.1±0.4 | 4.0±1.3 | **0.6±0.4** | **0.45±0.16** | 1.2±0.5 | **0.10±0.06** | **0.23±0.17** |
| ^10^Linalyl acetate | 2.2±0.6 | | **1.1±0.4** | **0.9±0.2** | 1.0±0.2 | **2.7±1.5** | **0.6±0.2** | 1.9±0.7 | **0.44±0.17** | **0.7±0.2** |
| ^11^(*Z*)-4-tert-Butylcyclohexyl  acetate | 2.8±0.9 | | **1.5±0.3** | 1.7±0.4 | 3.2±1.3 | 3.4±1.8 | 1.14±0.16 | 3.0±0.9 | **1.5±0.4** | **1.10±0.13** |
| ^12^α-Terpinyl acetate | 0.24±0.06 | | **0.19±0.05** | 0.19±0.06 | 0.24±0.07 | 0.35±0.10 | **0.13±0.04** | 0.34±0.11 | 0.23±0.13 | **0.10±0.02** |
| **Ketones** |  | |  |  |  |  |  |  |  |  |
| ^13^3-Methyl-2-butanone | 0.8±0.3 | | 1.7±0.9 | 0.33±0.14 | 6.9±2.1 | **3.0±0.9** | **1.1±0.3** | 2.6±1.0 | 3.5±1.4 | 3.1±2.2 |
| ^14^3-Pentanone | 2.9±0.4 | | 3.3±0.7 | **1.8±0.3** | 8.5±3.6 | **2.8±0.9** | 4.1±2.3 | 4.5±1.2 | 3.5±0.9 | 3.4±1.1 |
| ^15^3-Methyl-2-pentanone | 7.2±3.4 | | 7.0±3.1 | 1.7±0.5 | 30±17 | 6.9±3.1 | 1.5±0.3 | 10.8±4.2 | 6.6±1.8 | 9.3±5.4 |
| ^16^α-Isomethylionone | 0.8±0.3 | | 1.0±0.8 | 0.42±0.08 | 0.9±0.4 | 1.1±0.4 | 0.6±0.3 | 0.61±0.19 | 0.7±0.3 | 0.9±0.6 |
| **S- & N- containing compounds** |  | |  |  |  |  |  |  |  |  |
| ^17^3-Methylbutanenitrile | 4.9±1.6 | | 4.1±1.3 | 4.3±1.8 | 4.3±2.3 | **0.84±0.16** | **0.44±0.17** | 0.14±0.14 | 0.22±0.13 | 0.06±0.06 |
| ^18^Dimethyl disulfide | 4.4±1.3 | | 6.6±1.7 | **12.8±3.5** | 9.3±2.2 | 14±6.5 | 15±3.6 | 5.7±0.8 | **8.2±2.1** | **13.9±3.1** |
| ^19^Dimethyl trisulfide | 1.8±0.3 | | 2.2±0.5 | **3.8±1.4** | 4.2±1.3 | 4.0±1.9 | 3.8±0.8 | 3.0±0.6 | 2.7±0.4 | 2.9±0.6 |
| ^20^ 3-Butenyl isothiocyanate | 21.7±3.3 | | 41±17 | 86±60 | 10.1±3.9 | 24±15 | 19±11 | 123±51 | 83±36 | 140±39 |
| ^21^Benzyl nitrile | 38±15 | | 28.8±9.0 | 32±11 | 1.7±1.2 | 1.0±0.4 | 2.1±1.5 | 1.30±0.17 | **2.8±0.9** | **1.8±0.8** |
| ^22^Indole | 13.0±6.8 | | 12.3±2.8 | 7.6±3.6 | 5.9±2.0 | 6.2±2.4 | 3.1±0.9 | 16±11 | 7.0±3.1 | 9.5±4.8 |
| **Terpenoids** |  | |  |  |  |  |  |  |  |  |
| ^23^α-Thujene | 300±70 | | 314±58 | 290±71 | 155±58 | **97±56** | **87±34** | 448±60 | **304±85** | 400±100 |
| ^24^α-Pinene | 87±19 | | 91±16 | 84±21 | 54±15 | **34±17** | **31.5±7.9** | 125±14 | **83±19** | 103±24 |
| ^25^Sabinene | 681±137 | | 813±144 | 733±163 | 433±160 | **260±149** | **232±86** | 1179±157 | **681±189** | 1001±244 |
| ^26^β-Pinene | 57±13 | | 73±13 | 60±15 | 39±10 | 28±14 | **20.8±6.3** | 99±15 | **64±17** | 76±17 |
| ^27^β-Myrcene | 219±50 | | 236±43 | 215±50 | 126±46 | **86±42** | **68±25** | 336±46 | **220±62** | 311±80 |
| ^28^α-Terpinene | 42±23 | | **23.6±5.2** | **21.2±6.3** | 11.5±4.2 | **8.8±5.1** | **6.5±2.4** | 30.8±5.4 | 30.4±7.7 | 28.0±7.6 |
| ^29^Limonene | 310±73 | | 332±60 | 313±77 | 224±77 | **142±74** | **121±42** | 463±51 | **321±77** | 464±119 |
| ^30^1,8-Cineole | 79±19 | | 80±15 | 77±20 | 37±13 | **26±15** | **20.8±7.7** | 84±11 | 67±21 | 79±23 |
| ^31^(*E*)-β-Ocimene | 6.9±1.6 | | 7.4±1.4 | 6.5±1.7 | 4.1±1.3 | **2.8±1.5** | **2.1±0.9** | 9.6±1.6 | **6.2±1.5** | 8.7±2.4 |
| ^32^γ-Terpinene | 28±15 | | **16.3±3.5** | **14.9±4.4** | 7.9±2.8 | 6.2±3.5 | **4.7±1.7** | 21.5±3.7 | 20.8±5.1 | 19.7±5.2 |
| ^33^ (*E*)-β-Terpineol | 2.7±0.6 | | 3.1±0.5 | 3.2±0.9 | 1.8±0.7 | **1.0±0.6** | **0.9±0.4** | 4.3±0.6 | **2.2±0.7** | 3.7±1.0 |
| ^34^α-Terpinolene | 17.6±8.0 | | **11.5±2.5** | **10.4±3.0** | 5.7±2.0 | **4.3±2.4** | **3.2±1.1** | 15.5±2.4 | 13.4±3.1 | 13.6±3.5 |
| ^35^Linalool | 2.2±0.3 | | 2.3±0.5 | 2.6±0.6 | 1.8±0.7 | 1.0±0.4 | 0.7±0.3 | 4.1±0.7 | **2.4±0.9** | 4.1±1.2 |
| ^36^(*E*)-DMNT | 30.9±9.4 | | **92±53** | **7.9±2.4** | 210±180 | 19±9.6 | 15.6±3.9 | 2.8±0.9 | 1.9±0.6 | **17±13** |
| ^37^Menthol | 1.0±0.6 | | 0.46±0.15 | 0.6±0.2 | 0.58±0.12 | **7.0±6.0** | 0.46±0.16 | 0.47±0.16 | 0.9±0.6 | 0.44±0.14 |
| ^38^4-Terpineol | 0.64±0.16 | | 0.77±0.13 | 0.67±0.16 | 0.39±0.14 | 0.23±0.16 | **0.29±0.10** | 0.87±0.11 | **0.63±0.14** | **1.2±0.3** |
| ^39^α-Terpineol | 2.9±0.7 | | 6.2±2.4 | 2.8±1.2 | 3.3±2.1 | 1.5±1.0 | 1.2±0.6 | 9.1±3.1 | 4.6±2.4 | 5.2±2.3 |
| ^40^β-Cubebene | 0.07±0.03 | | **0.20±0.07** | 0.14±0.03 | 0.12±0.04 | 0.10±0.04 | 0.08±0.03 | 0.17±0.05 | **0.06±0.03** | **0.12±0.05** |
| ^41^β-Elemene | 17.2±7.9 | | 7.1±7.0 | **0.8±0.8** | 5.6±3.2 | 2.9±2.4 | 4.4±2.2 | 1.1±0.7 | **4.9±2.5** | 0.4±0.3 |
| ^42^Bicyclosesquiphellandrene | 0.17±0.05 | | **0.48±0.09** | 0.31±0.09 | 0.24±0.16 | 0.18±0.06 | 0.25±0.04 | 0.07±0.04 | 0.07±0.03 | **0.4±0.13** |
| ^43^(*Z*,*E*)-α-Farnesene | 1.7±0.6 | | 2.1±0.5 | 1.0±0.4 | 1.0±0.5 | 0.7±0.4 | 0.46±0.13 | 0.9±0.4 | 0.49±0.14 | 1.2±0.6 |
| ^44^β-Chamigrene | 2.4±1.1 | | **1.0±0.9** | **0.18±0.06** | 0.7±0.3 | 0.36±0.17 | 0.5±0.2 | 0.39±0.13 | **1.2±0.6** | **0.23±0.06** |
| ^45^(*E*,*E*)-α-Farnesene | 36±16 | | 54±21 | 28±11 | 28±19 | 20±12 | 8.0±3.2 | 21±11 | 18.5±5.7 | **40±22** |
| ^46^Germacrene A | 2.4±1.1 | | 1.2±1.1 | **0.17±0.12** | 0.6±0.3 | 0.5±0.4 | 0.6±0.3 | 0.10±0.04 | **0.7±0.4** | 0.07±0.03 |
| ^47^ (*Z*)-α-Bisabolene | 0.8±0.4 | | 0.4±0.4 | **0.04±0.04** | 0.25±0.16 | 0.14±0.12 | 0.19±0.09 | 0.08±0.05 | **0.27±0.12** | 0.02±0.01 |
| ^48^Nerolidol | 0.46±0.19 | | **0.64±0.18** | 0.65±0.12 | 1.4±0.3 | 1.6±0.7 | **0.7±0.3** | 0.5±0.2 | **0.16±0.07** | 0.37±0.17 |
| **other or unknown** |  | |  |  |  |  |  |  |  |  |
| ^49^4-Ethenyl cyclohexene | 2.9±1.4 | | **1.3±0.4** | 3.2±2.6 | 2.5±1.5 | 1.6±0.6 | 1.1±0.4 | 1.9±0.9 | 2.2±1.0 | **1.1±0.5** |
| ^50^Unknown | 0.8±0.2 | | **0.8±0.2** | 0.7±0.2 | 0.9±0.2 | 0.61±0.13 | 0.64±0.16 | 0.9±0.3 | 0.7±0.2 | 0.7±0.2 |
| ^51^Unknown | 2.0±0.5 | | **1.7±0.5** | **1.4±0.3** | 2.0±0.4 | 1.3±0.2 | **1.3±0.3** | 2.4±0.6 | **1.4±0.4** | **1.5±0.4** |
| Number of compounds that  increased in dually infested plants^a^ | | | 25 | 14 |  | 12 | 7 |  | 13 | 13 |
| Number of compounds that  decreased in dually infested plants^b^ | | | 26 | 37^**^ |  | 39^***^ | 44^***^ |  | 38^***^ | 38^***^ |

**Table 2** Summary of significance levels and parameters of OPLS-DA models comparing volatile emissions from plants with single and dual herbivore infestations

|  |  |  |  |  |  |  |  |
| --- | --- | --- | --- | --- | --- | --- | --- |
| Treatment comparisons between single and dual infestation | Cabbage population | Significant model? | Model type | PCs | R2X(cum) | R2Y(cum) | Q2(cum) |
| *P. xylostella* vs aphids (7d) + *P. xylostella^!^* | OH | No | OPLS-DA | 1+1+0 | 0.278 | 0.72 | -0.165 |
|  | KIM | No | OPLS-DA | 0+0+0 |  |  |  |
|  | WIN | No | OPLS-DA | 0+0+0 |  |  |  |
| *P. xylostella* vs aphids (14d) + *P. xylostella* | OH | No | OPLS-DA | 1+1+0 | 0.33 | 0.806 | -0.109 |
|  | KIM | Yes | OPLS-DA | 1+1+0 | 0.309 | 0.899 | 0.501 |
|  | WIN | No | OPLS-DA | 1+1+0 | 0.468 | 0.689 | 0.0868 |
| *M. brassicae* vs aphids (7d) + *M. brassicae* | OH | No | OPLS-DA | 1+1+0 | 0.371 | 0.819 | 0.19 |
|  | KIM | No | OPLS-DA | 1+1+0 | 0.457 | 0.524 | 0.0975 |
|  | WIN | No | OPLS-DA | 1+1+0 | 0.437 | 0.65 | -0.143 |
| *M. brassicae* vs aphids (14d) + *M. brassicae* | OH | No | OPLS-DA | 1+1+0 | 0.423 | 0.685 | 0.363 |
|  | KIM | No | OPLS-DA | 1+2+0 | 0.529 | 0.888 | 0.471 |
|  | WIN | No | OPLS-DA | 1+1+0 | 0.573 | 0.608 | 0.288 |

^!^Each cabbage population (*Brassica oleracea*: OH, KIM, WIN) was singly infested with 10 L2 *Plutella xylostella* or 30 L1 *Mamestra brassicae* caterpillars for 24 hours, or dually infested with both 20 adult aphids (*Brevicoryne brassicae*) for 7 / 14 days and either caterpillar species for 24 h.
